# The Anatomy of the Thymus Gland, with Numerous Plates

**Published:** 1845-02

**Authors:** 


*The Anatomy of the Thymus Gland, with numerous plates.* By SIR ASTLEY COOPER, etc. etc. etc. Royal Octavo. Philadelphia, Lea & Blanchard, 1845.

Few men in the profession have run so brilliant a career as the distinguished author of these treatises. Long the favourite of his Sovereign, he was scarcely less the idol of the profession, and of the elevated society among whom he exercised his calling. It is said, and probably with truth, that he received a larger amount of fees per annum from his individual patients than any other practitioner of the healing art, ancient or modern; and when we add to this fact the amount of hospital labour he performed, and the extent of his scientific investigations and literary performances, it is plain that his life was one of great industry, method, and devotion to his favourite pursuits. These traits of character are conspicuous in the productions now before us. They do not evince perhaps what many would call genius, but they exhibit to us models of careful examination and zealous inquiry. Whatever were the merits of Sir Astley as a Surgeon, and they were undoubtedly great, we are pursuaded that it is mainly on his anatomical labours that, in after times, his reputation as one of the ornaments of the profession must rest. The present edition, which embraces both treatises, in mechanical execution, is in good keeping with the work—good type and well leaded, thick white paper, beautiful lithographed illustrations—every think is as it ought to be, and in the highest degree creditable to the publishers.

